# Randomized Comparison of Terumo® Coated Slender™ versus Terumo® Noncoated Traditional Sheath during Radial Angiography or Percutaneous Coronary Intervention

**DOI:** 10.1155/2019/7348167

**Published:** 2019-03-04

**Authors:** Birthe Sindberg, Christel Gry Aagren Nielsen, Marianne Hestbjerg Poulsen, Martin Bøhme Rasmussen, Steen Carstensen, Troels Thim, Lars Jakobsen, Jacob Thorsted Sørensen, Benedicte Haastrup, Hanne Maare Søndergaard, Michael Mæng, Christian Juhl Terkelsen

**Affiliations:** ^1^Department of Cardiology, Aarhus University Hospital, DK-8200 Aarhus N, Denmark; ^2^Viborg Regional Hospital, Heibergs Alle 4, 8800 Viborg, Denmark

## Abstract

**Background:**

The transradial approach is generally associated with few complications. However, periprocedural pain is still a common issue, potentially related to sheath insertion and/or arterial spasm, and may result in conversion to femoral access. Radial artery occlusion (RAO) following the procedure is also a potential risk. We evaluate whether the design of the sheath has any impact on these variables.

**Methods:**

A total of 1,000 patients scheduled for radial CAG or PCI were randomized (1:1) to the use of a Slender or a Standard sheath during the procedure. Randomization was stratified according to chosen sheath size (5, 6, 7 French) and gender. A radial band was used to obtain hemostasis after the procedure, employing a rapid deflation technique. A reverse Barbeau test was performed to evaluate radial artery patency after removal of the radial band, and level of pain was assessed using a numeric rating scale (NRS).

**Results:**

Use of the Slender sheath was associated with less pain during sheath insertion (median NRS 1 versus 2, p=0.02), whereas no difference was observed in pain during the procedure, radial procedural success rates, use of analgesics and sedatives during the procedure, and radial artery patency following the procedure. Rate of RAO was 1.5% with no difference between groups.

**Conclusion:**

The use of the hydrophilic coated Slender sheath during radial CAG or PCI was associated with less pain during sheath insertion, whereas no difference in other endpoints was observed. A rapid deflation technique was associated with RAO of only 1.5%.

## 1. Introduction

The transradial approach has become increasingly popular worldwide when performing coronary angiography (CAG) or percutaneous coronary intervention (PCI) because of fewer bleeding complications [1]. There are, however, some limitations to the transradial approach. Periprocedural pain, potentially related to sheath insertion and/or arterial spasm, may result in conversion to femoral access. Furthermore, radial artery occlusion (RAO) following the procedure is a potential risk [2]. It is also unclear whether the design of the sheath has any impact on pain, rate of conversion to femoral access, or RAO following the procedure. Terumo (Terumo Corporation, Tokyo, Japan) has recently introduced a new kind of sheath, the Terumo® Glidesheath Slender™ (hereafter referred to as the “Slender sheath”). The sheath has a hydrophilic coating and is made of a thinner material than traditional sheaths, which gives it a smaller external diameter (6F = 2.46 mm) than a 6F Terumo Radiofocus sheath (= 2.62 mm) (hereafter referred to as the “Standard sheath”) [3].

The purpose of the present study was to evaluate whether the use of the 10 cm Slender sheath affects pain, rate of conversion to the femoral access, use of analgesics or sedatives, and RAO following the procedure compared to the 10 cm Standard sheath when performing radial CAG or PCI.

## 2. Methods

### 2.1. Study Population

**Figure 1:**
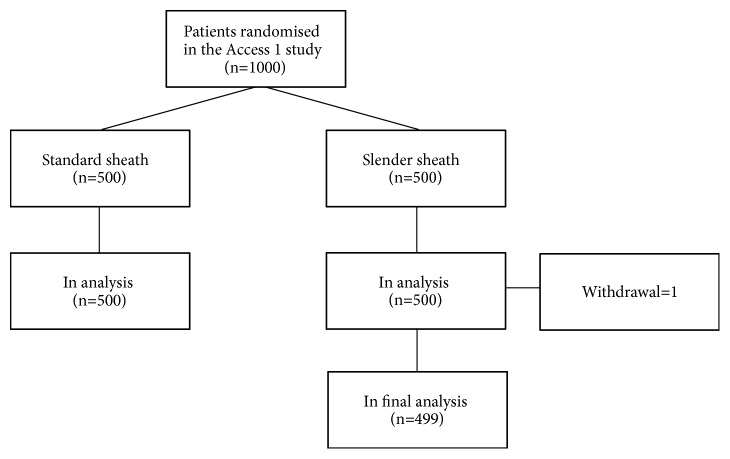
Flowchart.

ACCESS-1 was a randomized, controlled, prospective open-label two-center trial in which 1,000 patients we enrolled between the 15th of December 2015 and the 12th of July 2016 at Aarhus University Hospital Skejby, Aarhus N, Denmark and at Viborg Hospital, Denmark (Figure 1). Only patients aged ≥18 years were included. Exclusion criteria were abnormal Barbeau test (abnormal pulse oximetry findings on the thumb or index finger on sequential release of compression of ulnar and radial artery) or planned intervention using an 8F sheath. Operators having performed more than 50 radial procedures within the past 6 months were allowed to include patients.

Inclusion and randomization were performed in the catheterization laboratory before the procedure.

### 2.2. Randomization and Inclusion

Patients were randomly assigned (1:1), by a computerized IT system according to chosen sheath size (5, 6 or 7 French) and gender, to receive either a “Slender sheath” or a “Standard sheath” during radial CAG or PCI. Patients, investigators and clinicians were not masked to treatment allocation;however neither patients nor the nurses at the ward removing the TR-band and performing the reverse Barbeau test were told which treatment group the patients were randomized to.

### 2.3. Procedure

**Figure 2:**
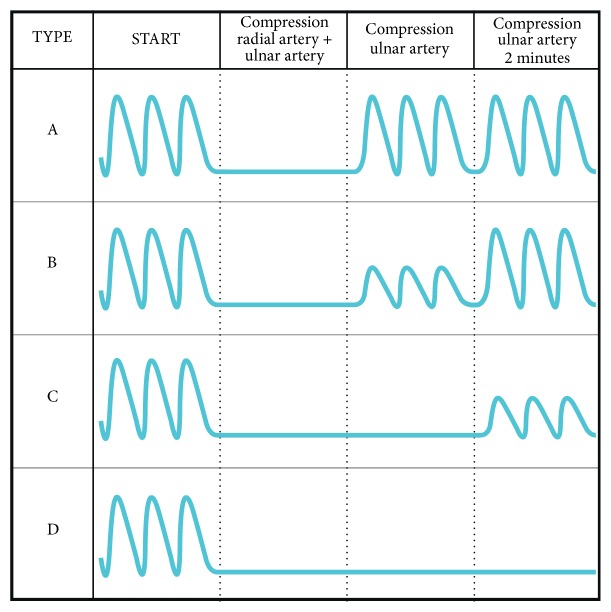
Reverse Barbeau test. Type A-D according to pulse oximetry findings after removal of the TR band at compression of a.radialis and a.ulnaris, or a.ulnaris alone.

Subcutaneous injection of Xylocaine 20mg/ml was used as local anesthesia at the access site. The sheath was inserted and the patients were asked to state their maximal perceived pain during sheath insertion according to a numeric rating scale (NRS) going from 0 to 10. The scales express subjective feelings (pain) as a numerical representation. We ensured the patient respond was related to the procedure of sheath insertion and not potential chest pain. While the NRS scale was well-known to the clinical staff, for consistency and to limit noise all nurses were re-trained in its use prior to study start. The training also took place during meetings, held routinely at the cath lab, and also together with the staff just prior to a CAG/PCI procedure along the full inclusion period. Prior to the procedure, 5000 IU heparin and 0.25 mg nitroglycerine were injected through the sheath. If PCI was performed, additional heparin was administered if found indicated by the physician. After the procedure, the patients were asked to state the maximal pain they had perceived during the procedure and during sheath removal according to the NRS. Following the procedure, a radial artery compression device (TR band) was applied. After initial inflation with 15 ml air, the TR band was slowly deflated until bleeding was observed. Approximately 1 ml air was then re-inflated to achieve hemostasis. The total air-volume inflated and the time of TR band application were recorded. At the ward, the nurses were instructed to deflate the TR band by removing 25% of the total air volume every 15 minutes (Rapid deflation technique). If bleeding occurred, the TR band was re-inflated with the volume removed, and the procedure was repeated after an additional 15 minutes. The time of TR band removal (time of hemostasis) was registered. Following removal of the TR band, a reverse Barbeau test was performed by the nurses at the ward to evaluate radial artery patency by monitoring pulse oximetry findings on the thumb or index finger when compressing the ulnar artery. The four different patterns of the reverse Barbeau test are illustrated in Figure 2. A reverse Barbeau type D was interpreted as presence of RAO. A physician experienced in arterial ultrasound performed an ultrasound evaluation and a reverse Barbeau test in a sample of 45 of the patients in whom the nurses had performed a test. This was done in order to qualify the accuracy of the reverse Barbeau tests performed by the nurses, and to evaluate the association between ultrasound findings and reverse Barbeau test findings.

All patients with reverse Barbeau D were scheduled for an outpatient visit after 1 month where the same physician would evaluate the radial artery patency with a reverse Barbeau test and do the arterial ultrasound examination.

To ensure standardization of the fitting and removal of the TR Band, we introduced the concept of a “contact nurse”. In each department one or two nurses were trained in the study protocol and agreed to ensure that their colleagues had sufficient knowledge about the study and were trained in the practical handling of the TR band and performance of the Reverse Barbeau test, to ensure consistency with data, throughout the study. Furthermore all study nurses meet regularly in each department both before study execution and during the study period.

### 2.4. Endpoints

The following primary endpoints were registered: (a) maximal pain (NRS) during sheath insertion, (b) maximal pain during the procedure (CAG or PCI) and during sheath removal (NRS), (c) proportion of patients converted to femoral access, (d) use of analgesics during the procedure (cumulated micrograms of fentanyl), (e) use of sedatives during the procedure (cumulated milligrams of midazolam), and (f) result of reverse Barbeau test after removal of the TR band.

Secondary endpoints were (a) number of catheters used and (b) number of sheaths used.

### 2.5. Ethical Consideration

The study was approved by the regional scientific ethical committee (J.nr.1-10-72-282-15) and the national data protection agency (J.nr. 1-16-02-679-15). The study was registered at ClinicalTrials.Gov (J.nr. NCT02637843). All patients provided written informed consent.

### 2.6. Statistical Analysis

Previous studies have indicated that patients who undergo radial PCI have an average pain score of 4 ± 2 (NRS), while it is 3 ± 2 for the radial CAG [4]. With alpha 0.05 and beta 0.80, 252 patients in each randomization arm were required to document a difference in NRS-indicated pain of 0.5 points, assuming that the average NRS-indicated pain is 3.5 with a spread of 2. For this reason we chose to randomize a total of 1,000 patients.

Data are analyzed according to the intention-to-treat principle. Continuous variables were presented as median (inter-quartile range, IQR) and comparisons were made using Mann-Whitney U test. Categorical variables were presented as proportions and compared using the Chi-square test. A p value <0.05 was considered statistically significant.

Statistical analyses were performed using STATA 14.0. (Stata Corporation, College Station, TX, USA).

## 3. Results

A total of 1,000 patients scheduled for subacute or acute CAG or PCI were included in this study of whom 891 patients were included at the regional PCI center (Aarhus University Hospital) and 109 patients at a satellite center performing only CAG (Viborg Regional Hospital) (Figure 1). Baseline characteristics (Table 1) were balanced. The number of patients treated using 5, 6, and 7 French sheaths were 128 (12.8%), 870 (87.1%), and 1 (0.1%), respectively. CAG only was performed in 655 (65.6%) and PCI in 344 (34.4%) patients, with no difference between the allocated treatment groups. A radial sheath was successfully inserted in 954 (95.5%) of the patients, and the radial procedure was completed without conversion to femoral access in 887 (88.8%). The number of included patients per operator ranged from 3 to 173. Successful sheath insertion varied from 67% (2/3) to 100% (21/21) and the radial success rate from also 67% (2/3) to 100% (9/9) among operators. Considering only the five operators including more than 100 procedures in the study, we found that successful sheath insertion varied from 93% (113/122) to 98% (151/154) and the radial success rate varied from 83% (101/122) to 95% (146/154). The nurses at the ward performed the reverse Barbeau test in 858 (90.0%) of the 954 patients who had a sheath inserted. The number of patients with reverse Barbeau results A was 692 (80.6%), result B 132 (15.3%), result C 21 (2.4%), and result D 13 (1.5%).

**Figure 3:**
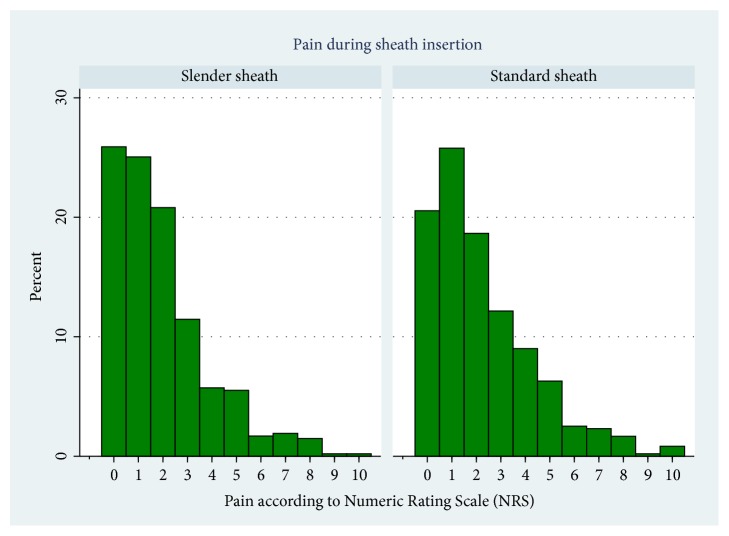
Pain during sheath insertion.

The primary and secondary endpoints are presented in Table 2. Use of the “Slender Sheath” was associated with significantly less pain during sheath insertion (median NRS 1 versus 2, P=0.02) (Figure 3), whereas no difference was observed in any of the remaining endpoints.

**Figure 4:**
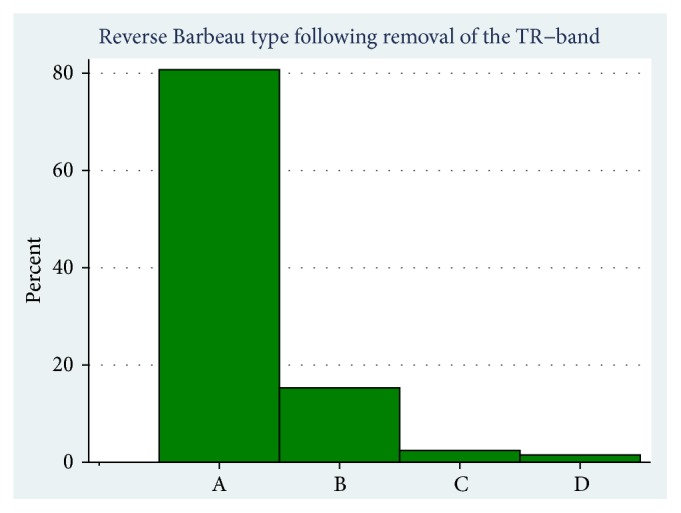
Results of Reverse Barbeau test.

Following removal of the TR band, 13 (1.5%) patients had a reverse Barbeau type D. (Figure 4). Eleven of these patients agreed to come to the 1-month follow-up, where they were offered an examination and ultrasound evaluation in the out-patient clinic. In seven patients, radial artery patency was achieved, with a reverse Barbeau type A observed in five patients and a Barbeau type C in two patients. Four patients still had a reverse Barbeau type D. None of these 11 patients had any clinical symptoms at 1-month follow-up.

In the 45 patients who had Reverse Barbeau test performed by both a nurse and a physician, there was agreement in the classified reverse Barbeau type in 40 (89%). In four patients, the physician evaluated reverse Barbeau type B and the nurse reverse Barbeau type A. In one patient, the physician evaluated reverse Barbeau type A and the nurse reverse Barbeau type B.

## 4. Discussion

It is well established that transradial access is associated with fewer complications, especially in patients with acute coronary syndromes [5, 6], and in elderly and high-risk patients [7]. However, tortuous arteries, arterial stenosis, and pain related to arterial spasm may prompt conversion to a femoral access. It seemed logical that smaller sheaths and coated sheaths would reduce pain and spasm tendency, which was the reason for performing the randomized comparison between the Slender and the Standard sheath. The main finding of less pain at insertion of the Slender sheath supports this hypothesis. However, the clinical impact of the findings may be limited, as there were no signs of reduced use of analgesics, sedatives, or less RAO when using the Slender instead of the Standard sheath. For this study we used the NRS scale to assess pain intensity during sheath insertion and removal. In previous studies the VAS scale has often been used. Even though VAS and NRS scales have been found to have good correlation; it is also stated that the NRS scale is more easy to use and that patients comply better to this instrument; plus the staff finds it easier to manage as there is no need for paper or a ruler [8, 9]. This is important as the patient during sheath insertion, and also during the procedure, cannot point at a scale as they are made sterile for the procedure. While it is worth noting that overall pain levels were lower than those reported in previous studies [4] there may be room for further reduction in pain levels during radial procedures. The use of analgesics and sedatives was restricted to around 20% and 25%, respectively, of patients in the present study. Analgesics and sedatives were administered only if the patient felt pain or discomfort. Future routine use of analgesics or sedatives upfront may reduce the pain levels even further as such use has been associated with a reduction in spasm tendency [10]. The use of verapamil was also very low in the present study (1-2%), but it is questionable whether increased use of verapamil would have any impact on pain [11]. Finally, the proportion of patients treated with 5 French sheaths was only around 13%, and a future increased use of 5 pieces of French equipment may be associated with even less procedural pain [12].

A second observation made in the present study was the extremely low rate of RAO in both groups, i.e., approximately 1.5% at discharge, with two-thirds even achieving patency at 1-month follow-up. This may be explained by the rapid deflation technique we deployed which aimed at removing the TR band as soon as possible. Notably, we did not install a fixed air volume, but instead deflated until bleeding occurred and then re-installed a volume of approximately 1 ml air. It seems logical that the optimal volume in the TR band varies from patient to patient, depending on how tight the TR-band is applied, and maybe the high rates of RAO observed in other studies can be explained by delayed initiation of deflation and inflation of fixed volumes [13]. More use of 5 French sheaths, or even sheath-less procedures, may further reduce RAO [14]. Previous studies show that high-dose heparin along with shorter compression times and patent hemostasis are associated with lower levels of RAO [15]. This is consistent with our findings. Even though the rate of RAO was low, the median time to removal of the TR band remained 125 minutes in the Slender sheath group and 129 minutes in the Standard sheath group. Whether it is possible to reduce time to hemostasis even further, without increasing RAO rates, warrants further evaluation and is the topic of the ongoing ACCESS-3 study (Oximetry guided versus traditional rapid deflation technique for achieving hemostasis after radial procedures, ClinicalTrials.gov ID NCT03626129).

## 5. Limitations

The rather high rate of conversions to femoral access may be explained by the fact that, historically, our center has prioritized femoral access, and we had just gradually switched to radial access within the past three years before the current study was conducted, at a time when many fellows were in training. It is well known that success rate is dependent on operator volume [16]. Nevertheless, all operators in the present study are high-volume operators in an international perspective given that the two centers perform 5,500 and 1,000 CAGs per year, respectively, and the PCI center also performs 2,800 PCI procedures per year. A 7 French sheath was used only in one patient; and even though there was limited clinical benefit of using a Slender sheath in the present study, we cannot exclude that the results would be different in patients scheduled for complex PCI using 7 French catheters. A separate study should evaluate this.

## 6. Conclusion

Use of a coated Slender sheath during radial CAG or PCI was associated with less pain during sheath insertion than use of a Standard sheath. No difference was observed in pain during the procedure, conversion to femoral access, use of analgesics, or radial artery patency following the procedure. A rapid deflation technique was associated with RAO of only 1.5%.

## Figures and Tables

**Table 1:** Baseline characteristics.

	Valid cases n=999	Slender sheath n=499	Standard sheath n=500	P
Age, years	999 (100%)	67.2 (57.9-74.0)	67.6 (59.3-74.9)	0.26

CAG vs. PCI	999 (100%)	65% vs. 35%	66% vs. 34%	0.77

Diabetes	904 (90%)	18.5%	19.3%	0.77

Hypertension	966 (97%)	61.2%	56.0%	0.11

Hyperlipidemia	971 (97%)	54.5%	59.8%	0.10

Previous MI	970 (97%)	18.4%	21.6%	0.21

Previous PCI	975 (98%)	22.6 %	25.8%	0.25

BMI	952 (95%)	27.2 (24.7-30.7)	26.8 (23.9-30.2)	0.13

Systolic BP, mmHg	999 (100%)	135 (120-157)	135 (120-155)	0.88

Diastolic BP, mmHg	999 (100%)	70 (60-80)	70 (60-80)	0.75

Procedural time, minutes				
All patients	996 (99.7%)	21.8 (10.9-39.3)	21.8 (10.9-35.0)	0.73
CAG alone		13.1 (8.7-24.0)	13.1 (8.7-24.0)	0.58
CAG+PCI		41.5 (28.4-61.2)	35.0 (26.2-52.4)	0.08

French size				0.60
5	128 (12.8%)	63 (13.0%)	65 (12.6%)	
6	870 (87.1%)	435 (87.0%)	435 (87.2%)	
7	1 (0.1%)	1 (0.2%)	0 (0 %)	

**Table 2:** Outcomes among patients treated with Slender versus Standard sheath during coronary angiography or percutaneous coronary intervention.

	Valid cases	Slender sheath	Standard sheath	P
Maximal pain, NRS				
During sheath insertion	947 (95%)	1 (0-3)	2 (1-3)	0.02
During the procedure	956 (96%)	2 (1-4)	2 (1-4)	0.30

No conversion to femoral access	999 (100%)	436 (87%)	451 (90%)	0.16

Use of verapamil	999 (100%)	5 (1.0%)	9 (1.8%)	0.28
Cumulated verapamil, mg.	5	5 (5-5)	5 (5-5)	

Use of analgesics	999 (100%)	109 (22%)	93 (19%)	0.20
Cumulated fentanyl, *μ*g.	202	50 (50-99)	50 (50-80)	0.92

Use of sedative	998 (99.9%)	132 (27%)	124 (25%)	0.54
Cumulated midazolam, mg.	256	1.5 (1.2-2.5)	1.5 (1.0-2.5)	0.92

Reverse Barbeau test	858 (86%)			0.52
A		342	350	
B		69	63	
C		13	8	
D		5	8	

Number of catheters used	997 (99.8%)	2 (2-3)	2 (2-3)	0.55

Number of sheaths used	996 (99.7%)	1 (1-1)	1 (1-1)	0.06

Time to hemostasis, min.	916 (91.7%)	125 (101-165)	129 (103-161)	0.96

## Data Availability

Anonymized data can be accessed by contacting the corresponding author with a protocol.

## Additional Points

*Impact on Daily Practice.* The present study does not support routine use of the Slender sheath instead of the Standard sheath but indicates that it may be of benefit in patients with a perceived high risk of spasm.
